# Gender- and age-dependent prevalence of malocclusions requiring orthodontic treatment according to the KIG classification. A cross-sectional study over a 20-year-period from the district of Viersen/North rhine

**DOI:** 10.1007/s00784-025-06607-8

**Published:** 2025-10-13

**Authors:** Gero Stefan Michael Kinzinger, Jan Hourfar, JörgAlexander Lisson

**Affiliations:** Department of Orthodontics, Saarland University, Homburg/Saar, 66424 Germany

**Keywords:** Malocclusion, Index system, KIG classification, Age, Gender

## Abstract

**Background and aim:**

Patients with statutory health insurance (SHI) in Germany must undergo an assessment of orthodontic treatment need using the KIG classification system since 2002. Recent studies have shown that the prevalence of anomalies is not homogeneous when categorised according to patient age at treatment begin. The aim of this study was to analyse the KIG classifications over a period of 20 years to determine whether there is also a gender-specific prevalence.

**Patients and methods:**

Between 2002 and 2021, *n* = 4940 statutorily insured patients before the age of 18 presented themselves for an orthodontic consultation. Out of these, *n* = 3701 showed a treatment indication according to current SHI guidelines. The KIG classification was based on the highest existing KIG grade observed in these patients without multiple classifications. The patient cohort was then divided into 3 groups according to gender and chronological age, representing early (PG 1), main (PG 2) and late (PG 3) treatment.

**Results:**

*N* = 1934 (52.2%, mean age 11.00 ± 2.28 years) of the patients were female, *n* = 1767 (47.8%, mean age 11.44 ± 2.23 years) male. Out of those, *n* = 1109 and thus 30.0% of the total collective had the KIG classification D and 43.1% (*n* = 1595) combined sagittal classifications D + M. Regarding treatment period, the classification K was the most frequent in PG 1, D in PG 2 and E in PG 3 for both genders, and the combination D + M in all groups. Regarding the age at treatment begin, there were different peak values for males and females. Females reached D + M, O + T, B + K and E + P as well as the KIG classifications D, M, O, T, B and K earlier than males, but even age distributions occurred for E and P. The percentage gender distribution in the various age groups showed the opposite trend: In females, there was a decrease in PG 3 compared to PG 1 for all 4 KIG combinations and for 7 of 8 individual anomalies, while it was vice versa in males.

**Conclusions:**

This study was the first to analyse the KIG classification for gender-specific differences and their possible age dependency over a period of 20 years and confirms that the distribution of KIG classifications requiring treatment is not homogeneous, but age-and gender-dependent.

**Clinical relevance:**

The study results on gender- and age-specific differences in the KIG classification can be regarded as a step towards personalised medicine in orthodontics.

## Introduction

In Germany, the eligibility for orthodontic treatment under the statutory health insurance scheme (GKV) was restricted on 1 January 2002 [1]. Patients with statutory health insurance are only entitled to orthodontic treatment if there are medically justified classifications of a certain degree of severity (KIG: »**K**ieferorthopädische **I**ndikations**g**ruppen« – orthodontic indication groups, classification and grades, Table 1). Over the last 20 years, only three single-session cross-sectional studies have been conducted in various regions of Germany on selected patient groups using the KIG system to evaluate, among other things, the frequency of anomalies and the need for orthodontic treatment [2–6].

**Table 1:** Kieferorthopädische Indikationsgruppen« (KIG; orthodontic indication groups) according to the guidelines of the federal committee of dentists and health insurance funds for orthodontic treatment (figures in mm)

KIG classification	Description	Grade 1	Grade 2	Grade 3	Grade 4	Grade 5
A	Craniofacial anomalies	-	-	-	-	(Cleft palate and syndromes)
U	Missing teeth (Agenesis or loss)	-	-	-	missing teeth	-
S	Eruption disorders	-	-	-	Impaction (except for third molars)	displacement (except for third molars)
D	**Sagittal discrepancy ** **increased overjet**	**≤ 3**	**> 3 ≤ 6**	**-**	> 6 ≤ 9	> 9
M	**Sagittal discrepancy ** **negative overjet**	**-**	**-**	**-**	0 ≤ 3	> 3
O	**Vertical discrepancy open bite**	**≤ 1**	**> 1 ≤ 2**	> 2 ≤ 4	> 4 habitually open	> 4 skeletally open
T	**Vertical discrepancy deep bite**	**> 1 ≤ 3**	**> 3** **with/without mucosal contact**	> 3 with traumatic mucosal impingement	-	-
B	**Transverse discrepancy scissors bite**	**-**	**-**	-	Scissors bite	-
K	**Transverse discrepancy crossbite**	**-**	**Buccolingually cusp-to-cusp relation**	Bilateral crossbite	unilateral crossbite	-
E	**Contact point displacement**	**< 1**	**> 1 ≤ 3**	> 3 ≤ 5	> 5	-
P	**Space deficiency**	**-**	**≤ 3**	> 3 ≤ 4	> 4	-

In addition to the single-session cross-sectional studies, four recent studies [7–10] evaluated the frequency and long-term distribution of individual malocclusions at initial consultation in a regional population with different study questions. In contrast to the previous single-session cross-sectional studies on selected patient groups, all KIG occurrences were recorded as part of long-term studies in an unselected clientele of a private orthodontic practice [7, 9, 10].

Over a period of 20 years (2002–2021), *n* = 1,234 patients (24.98% of *n* = 4,940) presented with KIG classification D (increased overjet). Of the 11 KIG classifications, 86.52% were among the six most frequent classifications (D, E, K, S, P and M), while only 13.49% were among the five rarest classifications (U, B, T, O and A). This distribution pattern of KIG classifications remained consistent across four separately investigated 5-year periods. The sagittal classifications D and M always were the most common treatment indications [7].

Comparing regionally acquired data with the DMS•6 results and KZBV billing information from 2020 showed that sagittal classifications D and M accounted for 47.4% of malocclusions requiring therapy, with KIG grade D4 being the most common anomaly. The representativeness of the practice data was also proven. There were no regional deviations in the prevalence of KIG grades 3–5 requiring treatment from the national average, even over a longer observation period [9].

Regarding age at treatment begin, statutorily insured patients showed an age-dependent prevalence in an investigation over a 10-year period between 2012 and 2021: The distribution of KIG classifications requiring treatment was not homogeneous. The differences were particularly evident in the early treatment group. This is probably because the KIG classification is by statutory regulations not fully applicable to patients before the second mixed dentition phase. Still, sagittal classifications (D + M) were most frequent across all age groups. The KIG classification D was the most frequent anomaly up to the age of 18 [10].

It has not yet been investigated if the distribution of KIG classifications in minors over a 20-year period regarding spatial plane and abnormal tooth position is also gender-dependent. It is unclear whether certain classifications are generally more common in male or female patients or only occur at a certain age.

### Aim of the study

The study aimed.

1. to determine whether malocclusions in minors exhibit gender-dependent distribution patterns in terms of spatial plane and abnormal tooth position, and.
2. to determine whether there are gender-dependent differences that only occur at certain ages.

over a long observation period.

### Patients and methods

In a twenty-year period between 2002 and 2021, *n* = 4940 statutorily insured patients presented themselves in a private practice in the district of Viersen/North Rhine, Germany, for an initial consultation and thus for KIG classification and eligibility for orthodontic treatment.

*N* = 3701 patients up to the age of 18 showed eligibility in 8 KIG classifications D, M, O, T, B, K, E and P with KIG-grades 3, 4 or 5. Only patients with KIG-grade ≥ 3 were eligible for orthodontic treatment provided through the statutory health insurance.

The study cohort was divided into 3 patient groups (PG) for analysis according to gender (female/male) and chronological age:

- PG 1 < 10 years of age (early Tx),
- PG 2 10-<13 years of age (main Tx) and.
- PG 3 13-<18 years of age (late Tx).

The chosen intervals for the patient groups reflect treatment phases in different stages within the development of the dentition, i.e. early mixed dentition, mixed dentition and permanent dentition, in accordance with descriptions in previous studies [11, 12].

### KIG system: classifications

In this study, all malocclusions were categorised into 8 of 11 possible classifications of the KIG indication system. The shown order represents the classification ranking demanded by the statutory health system:

D Sagittal discrepancy increased overjet.

M Sagittal discrepancy negative overjet.

O Vertical discrepancy open bite (habitually open/skeletally open).

T Vertical discrepancy deep bite (with/without mucosal contact; with traumatic mucosal impingement.

B Transverse discrepancy scissors bite.

K Transverse discrepancy crossbite (Buccolingually cusp-to-cusp relation, Bilateral crossbite, Unilateral crossbite).

E Contact point displacement.

P Space deficiency.

KIG classifications and grades were recorded through clinical inspection only and validated exclusively by orthodontists, who applied the four-eye principle throughout. This is in accordance with the current legislation for standard, non-surgical cases. The extent of overjet, overbite, crowding and space deficits were measured intraorally using sliding callipers »Münchner Modell^®^« (Dentaurum, Ispringen, Germany) with a precision of 0.25 mm. The occlusion regarding frontal and lateral crossbites was assessed visually.

The classifications D + M record sagittal, O + T vertical, and B + K transverse discrepancies. E + P represent tooth position anomalies. The also existing KIG classifications A, S and U were not recorded since these require additional x-ray examination.

The KIG grades 3–5 requiring treatment according to the valid statutory health system guidelines [1] were categorised only into the highest of 19 possible combinations of classification and grade as required by the statutory regulations. Since only the highest classification and grade were recorded, multiple findings per patient were impossible.

### Statistical analysis

Anonymized patient data was collated using a spread sheet software (Excel^®^, Microsoft Corp., Redmond, WA, USA). Normal distribution of the variable “age” was evaluated graphically and using the Shapiro-Wilk-Test with SPSS^®^ Version 28 for Windows^®^ (IBM Corp., Armonk, NY, USA). Mean and standard deviation was reported for the latter variable. All other data was interpreted descriptively.

## Results

### Patient age and gender distribution of statutorily insured patients over a 20-year period (Fig. 1; Tables 2 and 3)

**Fig. 1:**
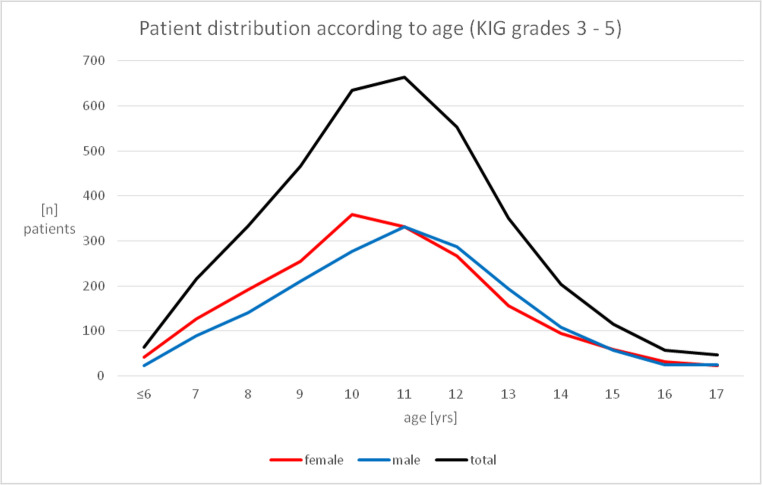
Frequency and age distribution of n=3701 statutorily insured patients distributed among 8 investigated KIG classifications D, M, O, T, B, K, E and P between 2002 and 2021

Out of *n* = 3701 patients, *n* = 1934 (52.2%) were female, and *n* = 1767 (47.8%) were male. At the initial consultation, the average age of the females was 11.09 ± 2.28, and that of the males 11.44 ± 2.23 years. The age distribution showed a peak for all patients between 9 and 12 years, with highest values at 10 and 11: KIG classification was mainly performed around 10 years in females and around 11 years in males (Fig. 1; Table 2).

**Table 2:** Age and gender distribution of *n* = 3701 statutorily insured patients between 2002 and 2021 with initial orthodontic consultation before the age of 18, and with KIG classifications D, M, O, T, B, K, E and P (grades 3, 4 and 5)

KIG classification grades 3–5	Gender	Distri- bution [*n*]	Mean patient age [years]	Patient distribution according to age (years) [*n*]											
			M ± SD	≤ 6	7	8	9	10	11	12	13	14	15	16	17
D	female	566	11.10 ± 1.87	1	20	32	101	147	120	71	33	16	14	6	5
	male	543	11.53 ± 1.83	2	13	23	63	110	137	102	50	18	11	8	6
	total	1109	11.31 ± 1.86	3	33	55	164	257	257	173	83	34	25	14	11
M	female	259	10,27 ± 2.62	18	37	47	31	31	32	20	16	14	5	6	2
	male	227	10.78 ± 2.57	7	26	26	40	29	31	25	15	10	11	3	4
	total	486	10.51 ± 2.61	25	63	73	71	60	63	45	31	24	16	9	6
O	female	35	11.36 ± 2.22	0	0	3	9	6	7	1	4	2	2	0	1
	male	32	12.21 ± 2.73	0	1	3	2	9	2	4	2	3	3	0	3
	total	67	11.77 ± 2.49	0	1	6	11	15	9	5	6	5	5	0	4
T	female	42	12.02 ± 1.75	0	0	0	4	11	11	4	5	5	1	1	0
	male	56	12.10 ± 1.79	0	1	2	3	6	13	14	12	2	1	2	0
	total	98	12.07 ± 1.77	0	1	2	7	17	24	18	17	7	2	3	0
B	female	99	12.68 ± 1.79	0	1	2	3	9	15	29	18	12	7	1	2
	male	106	13.02 ± 1.80	0	0	3	3	5	16	23	26	18	7	3	2
	total	205	12.86 ± 1.80	0	1	5	6	14	31	52	44	30	14	4	4
K	female	337	10.62 ± 2.74	18	45	55	41	29	41	37	30	14	14	8	5
	male	275	10.78 ± 2.67	11	33	43	39	24	29	31	30	19	7	5	4
	total	612	10.69 ± 2.71	29	78	98	80	53	70	68	60	33	21	13	9
E	female	316	12.23 ± 1.83	0	1	9	13	59	67	70	44	28	13	7	5
	male	238	12.63 ± 1.84	1	0	6	9	22	48	57	40	33	14	3	5
	total	554	12.40 ± 1.84	1	1	15	22	81	115	127	84	61	27	10	10
P	female	280	10.36 ± 1.89	4	22	44	53	67	39	34	6	4	2	3	2
	male	290	10.65 ± 1.74	1	16	35	52	71	56	31	19	5	3	1	0
	total	570	10.50 ± 1.82	5	38	79	105	138	95	65	25	9	5	4	2
all 8	female	1934	11.09 ± 2.28	41	126	192	255	359	332	266	156	95	58	32	22
	male	1767	11.44 ± 2.23	22	90	141	211	276	332	287	194	108	57	25	24
	total	3701	11.26 ± 2.26	63	216	333	466	635	664	553	350	203	115	57	46

Among the defined treatment period, the patient distribution was as follows (Table 3):

**Table 3:** Frequency, percentage and gender distribution of the 8 KIG classifications (grades 3, 4, and 5) in PG1, PG2 and PG3 before the age of 18 between 2002 and 2021

KIG classification grades 3–5	Gender	Distribution	< 10. yrs	< 10. yrs	10.−13. yrs	10.−13. yrs	> 13.−18. yrs	> 13.−18. yrs
		n	n	%	n	%	n	%
D	female	566	154	60.4	338	49.2	74	44.3
	male	543	101	39.6	349	50.8	93	55.7
	total	1109	255	100	687	100	167	100
M	female	259	133	57.3	83	49.4	43	50.0
	male	227	99	42.7	85	50.6	43	50.0
	total	486	232	100	168	100	86	100
O	female	35	12	66.7	14	48.3	9	45.0
	male	32	6	33.3	15	51.7	11	55.0
	total	67	18	100	29	100	20	100
T	female	42	4	40.0	26	44.1	12	41.4
	male	56	6	60.0	33	55.9	17	58.6
	total	98	10	100	59	100	29	100
B	female	99	6	50.0	53	54.6	40	41.7
	male	106	6	50.0	44	45.4	56	58.3
	total	205	12	100	97	100	96	100
K	female	337	159	55.8	107	56.0	71	52.2
	male	275	126	44.2	84	44.0	65	47.8
	total	612	285	100	191	100	136	100
E	female	316	23	59.0	196	60.7	97	50.5
	male	238	16	41.0	127	39.3	95	49.5
	total	554	39	100	323	100	192	100
P	female	280	123	54.2	140	47.0	17	37.8
	male	290	104	45.8	158	53.0	28	62.2
	total	570	227	100	298	100	45	100
all 8	female	1934	614	57.0	957	51.7	363	47.1
	male	1767	464	43.0	895	48.3	408	52.9
	total	3701	1078	100	1852	100	771	100

- PG 1: *n* = 1078 patients, *n* = 614 (57.0%) female, *n* = 464 (43.0%) male, early treatment,
- PG 2: *n* = 1852 patients, *n* = 957 (51.7%) female, *n* = 895 (48.3%) male, main treatment,
- PG 3: *n* = 771 patients, *n* = 363 (47.1%) female, *n* = 408 (52.9%) male, late treatment.

These figures showed that girls mainly received early or main treatment, whereas boys mainly received main or late treatment.

### Frequency, age and gender distribution for the KIG classifications D, M, O, T, B, K, E and P with KIG grades 3–5 (Fig. 2a-h; Table 2)

#### All patients: frequency of KIG classifications (Fig. 2a-h; Table 2)

Over the 20-year-period, *n* = 1109 (30.0%) patients showed KIG classification D. Classifications K (*n* = 612, 16.6%), P (*n* = 570, 15.4%), E (*n* = 554, 15.0%) and M (*n* = 486, 13.1%) occurred in more than 10%., whereas classifications B (*n* = 205, 5.5%), T (*n* = 98, 2.6%) and O (*n* = 67, 1.8%) were recorded less frequently.

#### Gender distribution in KIG classifications D, M, O, T, B, K, E and P (Fig. 2a-h; Table 2)

Female patients predominate in 5 classifications. D: *n* = 566 female/*n* = 543 male, K: *n* = 337 female/*n* = 275 male, E: *n* = 316 female/*n* = 238 male, M: *n* = 259 female/*n* = 227 male, O: *n* = 35 female/*n* = 32 male.

Male patients predominate in 3 classifications. P: *n* = 290 male/*n* = 280 female, B: *n* = 106 male/*n* = 99 female, T: *n* = 56 male/*n* = 42 female.

#### Age distribution in KIG classifications D, M, O, T, B, K, E and P (Fig. 2a-h; Table 2)

**Fig. 2:**
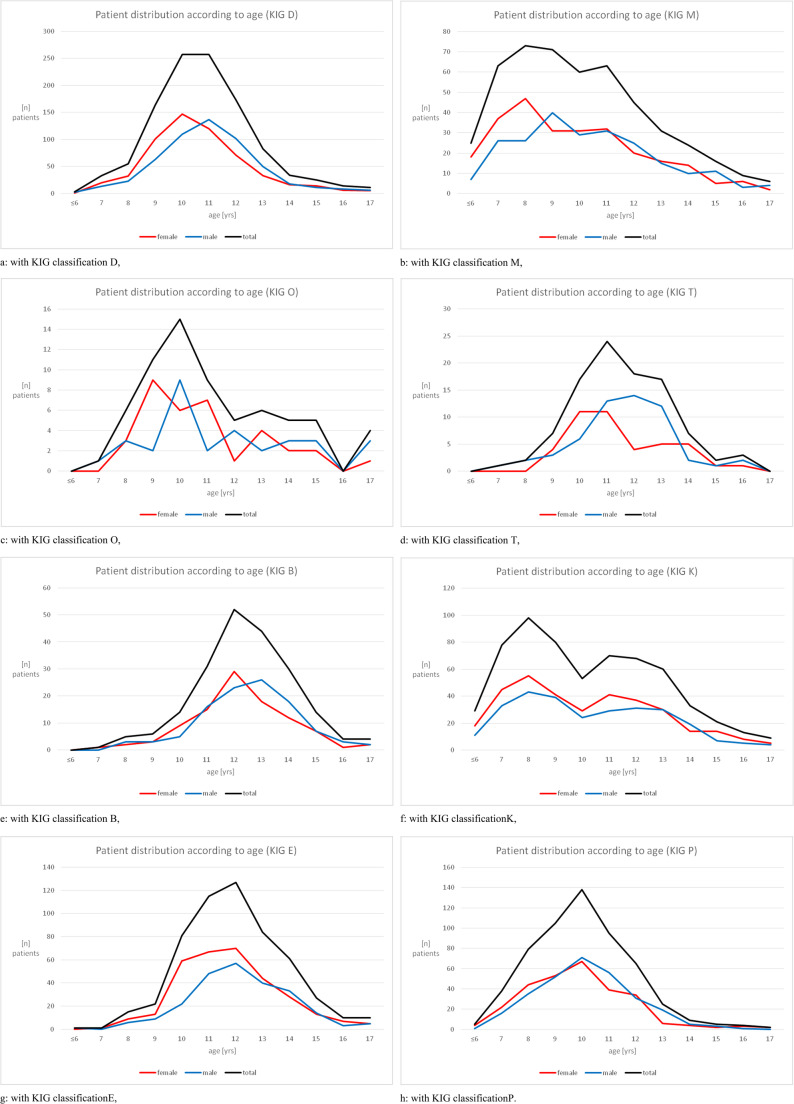
Frequency and age distribution of patients between 2002 and 2021, broken down by KIG classification (all patients, male and female patients)

The age distribution in the classification D shows an age peak at 10 and 11 years, with high values for female patients at 10 years and for male patients at 11 years (Fig. 2a).

In classification M, most patients were diagnosed between 8 and 9 years (females at 8, males at 9 years). There is a slight increase at the age of 11 years (Fig. 2b). Classification O was mainly recorded between 9 and 10 years, with female patients at 9 and male patients at 10 years (Fig. 2c). In classification T, patient age ranged from 10 to 12 years (female patients at 10 and 11, male patients at 11 and 12 years) (Fig. 2d). In patients with classification B, an age peak was recorded for 12 years in female and 13 years in male patients (Fig. 2e). The age distribution in the classification K showed two peaks at 8 and 11 years of age in the total group and in female patients, and at 8 years and 12 or 13 in male patients (Fig. 2f). All patients with the classifications E and P showed peak distributions for both genders at 12 years for E (Fig. 2g) and at 10 years for P (Fig. 2h).

Females were always diagnosed earlier with KIG classifications D, M, O, T, B and K, with classification K presenting an additional 2nd age peak for initial diagnosis. There was no gender-or age-specific difference for classifications E and P.

### Frequency and gender distribution by treatment period in KIG classifications D, M, O, T, B, K, E and P within patient groups 1–3 in a 20-year-period (Table 3)

#### PG 1 – Early treatment (Table 3)

Out of *n* = 1078 patients in PG 1, *n* = 285 (26.4%) presented classification K, *n* = 255 (23.6%) D, *n* = 232 (21.5%) M and *n* = 227 (21.2%) P, thus representing 92.7% of all findings. The remaining 7.3% were allocated to classifications E (*n* = 39, 3.6%), O (*n* = 18, 1.7%) B (*n* = 12, 1.1%), and T (*n* = 10, 0.9%).

Female patients predominated both the entire and – with the exception T – the classification groups: K: *n* = 159 f (55.8%)/*n* = 126 m (44.2%), D: *n* = 154 f (60.4%)/*n* = 101 m (39.6%), M: *n* = 133 f (57.3%)/*n* = 99 m (42.7%), P: *n* = 123 f (54.2%)/*n* = 104 m (45.8%), E: *n* = 23 f (59.0%)/*n* = 16 m (41.0%), and O: *n* = 12 f (66.7%)/*n* = 6 m (33.3%). Males were more frequent in classification T: *n* = 6 m (60%)/*n* = 4 f (40%).

The percentage differences were thus particularly obvious for classifications D, O and T. In B, the gender ratio is balanced: 6 female/6 male (50% each).

#### PG 2 – Main treatment (Table 3)

Out of *n* = 1852 patients in PG 2, *n* = 687 (37.1%) presented classification D, *n* = 323 (17.4%) E, *n* = 298 (16.1%) P, and *n* = 191 (10.3%) K, thus representing 80.9% of all findings. The remaining 19.1% were allocated with less that 10% each to classifications M (*n* = 168, 9.1%), B (*n* = 97, 5.2%), T (*n* = 59, 3.2%) and O (*n* = 29 patients, 1.6%).

Female patients predominated the entire and three classification groups: E: *n* = 196 f (60.7%)/*n* = 127 m (39.3%), K: *n* = 107 f (56.0%)/*n* = 84 m (44.0%), B: *n* = 53 f (54.6%)/*n* = 44 m (45.4%). Males were more frequent in classifications D: *n* = 349 m (50.8%)/*n* = 338 f (49.2%), P: *n* = 158 m (53.0%)/*n* = 140 f (47.0%), M *n* = 85 m (50.6%)/*n* = 83 f (49.4%), T *n* = 33 m (55.9%)/*n* = 26 f (44.1%), and O *n* = 15 m (51.7%)/*n* = 14 f (48.3%). The gender ratio differed markedly for E and appeared almost balanced in the other classifications.

#### PG 3 – Late treatment (Table 3)

Out of *n* = 771 patients in PG3, *n* = 192 (24.9%) showed classification E, *n* = 167 (21.7%) D, *n* = 136 (17.6%) K, *n* = 96 (12.4%) B and *n* = 86 (11.2%) M, thus representing 87.8% of all findings. The remaining 12.2% were allocated with less that 10% each to classifications P (*n* = 45, 5.8%), T (*n* = 29, 3.8%) and O (*n* = 20, 2.6%) accounted for less than 10%.

Male patients predominated the entire and five classification groups: D *n* = 93 m (55.7%)/*n* = 74 f (44.3%), B *n* = 56 m (58.3%)/*n* = 40 f (41.7%), P *n* = 28 m (62.2%)/*n* = 17 f (37.8%), T *n* = 17 m (58.6%)/*n* = 12 f (41.4%), O *n* = 11 m (55.0%)/*n* = 9 f(45.0%). Females were more frequent in classifications E *n* = 97 f (50.5%)/*n* = 95 m (49.5%) and K *n* = 71 f (52.2%)/*n* = 65 m (47.8%). Classification M showed an equal gender ratio: n = 43 f/*n* = 43 m (50% each).

The gender ratio only differed markedly in classification P.

### Percentage comparison of gender-specific allocation of KIG classifications in PG 1, 2 and 3 (Fig. 3a and b; Table 3)

**Fig. 3:**
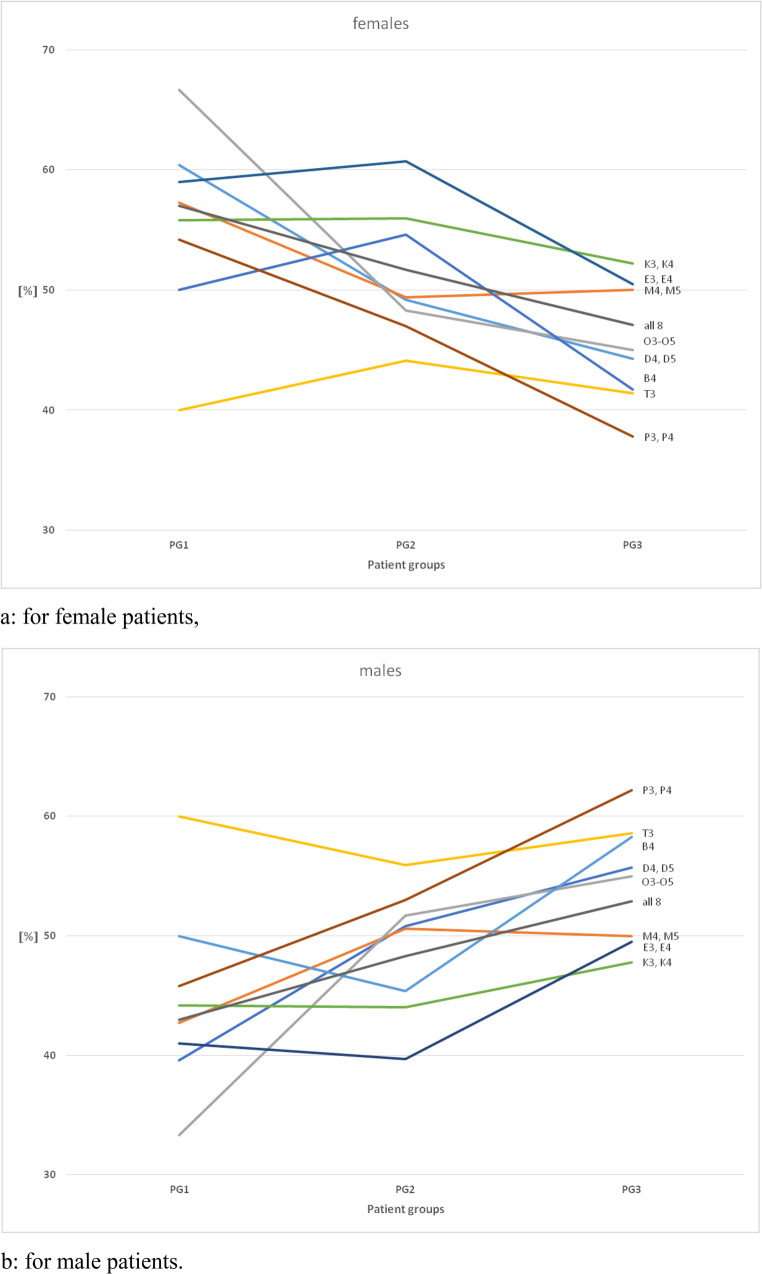
Comparison of the frequency of the 8 different KIG classifications with grades 3–5 in PG 1, PG 2 and PG 3 between 2002-2021

Figures 3a and b visualise the contrasting gender distribution across the different age groups:

In female patients, a decrease in PG 3 compared with PG 1 is observed in all patients and in 7 out of 8 classifications, with a nearly linear course in the overall patients and in the classifications D, O and P, with a medium high in PG 2 in B, K and E and a medium low in PG 2 in M. In the female patients, a decrease in PG 3 compared with PG 1 is observed in all patients and in 7 out of 8 classifications.

Contrary to that, there is an increase in the percentage of male patient involvement in PG 3 compared to PG 1 for all patients and for 7 out of 8 classifications, whereby the progression is also almost linear for the overall patients and classifications D, O and P, with an intermediate low in PG 2 for B, K and E and an intermediate high in PG 2 for M.

### Frequency and gender distribution of KIG classifications regarding spatial plane and abnormal tooth position (Fig. 4a-d; Tables 4 and 5)

**Table 4:** Age and gender distribution of *n* = 3701 statutorily insured patients between 2002 and 2021 with initial orthodontic consultation before the age of 18, and with distribution according to Spatial planes and tooth position anomalies (KIG classifications D + M, O + T, B + K, E + P with grades 3–5)

Combined KIG classification grades 3–5	Gender	Distri- bution [*n*]	Mean patient age [years]	Patient distribution according to age (years) [*n*]											
			M ± SD	≤ 6	7	8	9	10	11	12	13	14	15	16	17
D + M (sagittal)	female	825	10.84 ± 2.17	19	57	79	132	178	152	91	49	30	19	12	7
	male	770	11.31 ± 2.11	9	39	49	103	139	168	127	65	28	22	11	10
	total	1595	11.07 ± 2.15	28	96	128	235	317	320	218	114	58	41	23	17
O + T (vertical)	female	77	11.72 ± 1.99	0	0	3	13	17	18	5	9	7	3	1	1
	male	88	12.14 ± 2.16	0	2	5	5	15	15	18	14	5	4	2	3
	total	165	11.95 ± 2.09	0	2	8	18	32	33	23	23	12	7	3	4
B + K (transverse)	female	436	11.09 ± 2.70	18	46	57	44	38	56	66	48	26	21	9	7
	male	381	11.41 ± 2.65	11	33	46	42	29	45	54	56	37	14	8	6
	total	817	11.24 ± 2.68	29	79	103	86	67	101	120	104	63	35	17	13
E + P (tooth malposition)	female	596	11.35 ± 2.08	4	23	53	66	126	106	104	50	32	15	10	7
	male	528	11.54 ± 2.04	2	16	41	61	93	104	88	59	38	17	4	5
	total	1124	11.44 ± 2.06	6	39	94	127	219	210	192	109	70	32	14	12
all 4 combined	female	1934	11.09 ± 2.28	41	126	192	255	359	332	266	156	95	58	32	22
	male	1767	11.44 ± 2.23	22	90	141	211	276	332	287	194	108	57	25	24
	total	3701	11.26 ± 2.26	63	216	333	466	635	664	553	350	203	115	57	46

**Table 5:** Frequency, percentage and gender distribution with distribution according to Spatial planes and tooth position anomalies (KIG classifications D + M, O + T, B + K, E + P with grades 3–5) in PG1, PG2 and PG3 before the age of 18 between 2002 and 2021

Combined KIG-classifications grades 3–5	Gender	Distribution	< 10 yrs	< 10 yrs	10–13 yrs	10–13 yrs	> 13–18 yrs	> 13–18 yrs
		n	n	%	n	%	n	%
D + M	female	825	287	58.9	421	49.2	117	46.2
	male	770	200	41.1	434	50.8	136	53.8
	total	1595	487	100	855	100	253	100
O + T	female	77	16	57.1	40	45.5	21	42.9
	male	88	12	42.9	48	54.5	28	57.1
	total	165	28	100	88	100	49	100
B + K	female	436	165	55.6	160	55.6	111	47.8
	male	381	132	44.4	128	44.4	121	52.2
	total	817	297	100	288	100	232	100
E + P	female	596	146	54.9	336	54.1	114	48.1
	male	528	120	45.1	285	45.9	123	51.9
	total	1124	266	100	621	100	237	100
all 4 combined	female	1934	614	57.0	957	51.7	363	47.1
	male	1767	464	43.0	895	48.3	408	52.9
	total	3701	1078	100	1852	100	771	100

#### Total collective (n = 3701): distribution regarding Spatial plane and abnormal tooth position (Table 4)

The sagittal deviations D + M represented 43.1% (*n* = 1595), the vertical deviations O + T 4.4% (*n* = 165), the transverse deviations B + K 22.1% (*n* = 817) of the anomalies eligible for treatment. The tooth position anomalies E + P reached a frequency of 30.4% (*n* = 1124).

#### Gender distribution regarding Spatial plane and abnormal tooth position (Table 4)

Female patients predominated most classifications: sagittal D + M (*n* = 825 f/*n* = 770 m), transverse B + K (*n* = 436 f/*n* = 381 m) and tooth position E + P (*n* = 596 f/*n* = 528 m). Contrary to that, male patients showed more vertical deviations: O + T (*n* = 88 m/*n* = 77 f).

#### Age distribution regarding Spatial plane and abnormal tooth position (Fig. 4a-d; Table 4)

**Fig. 4:**
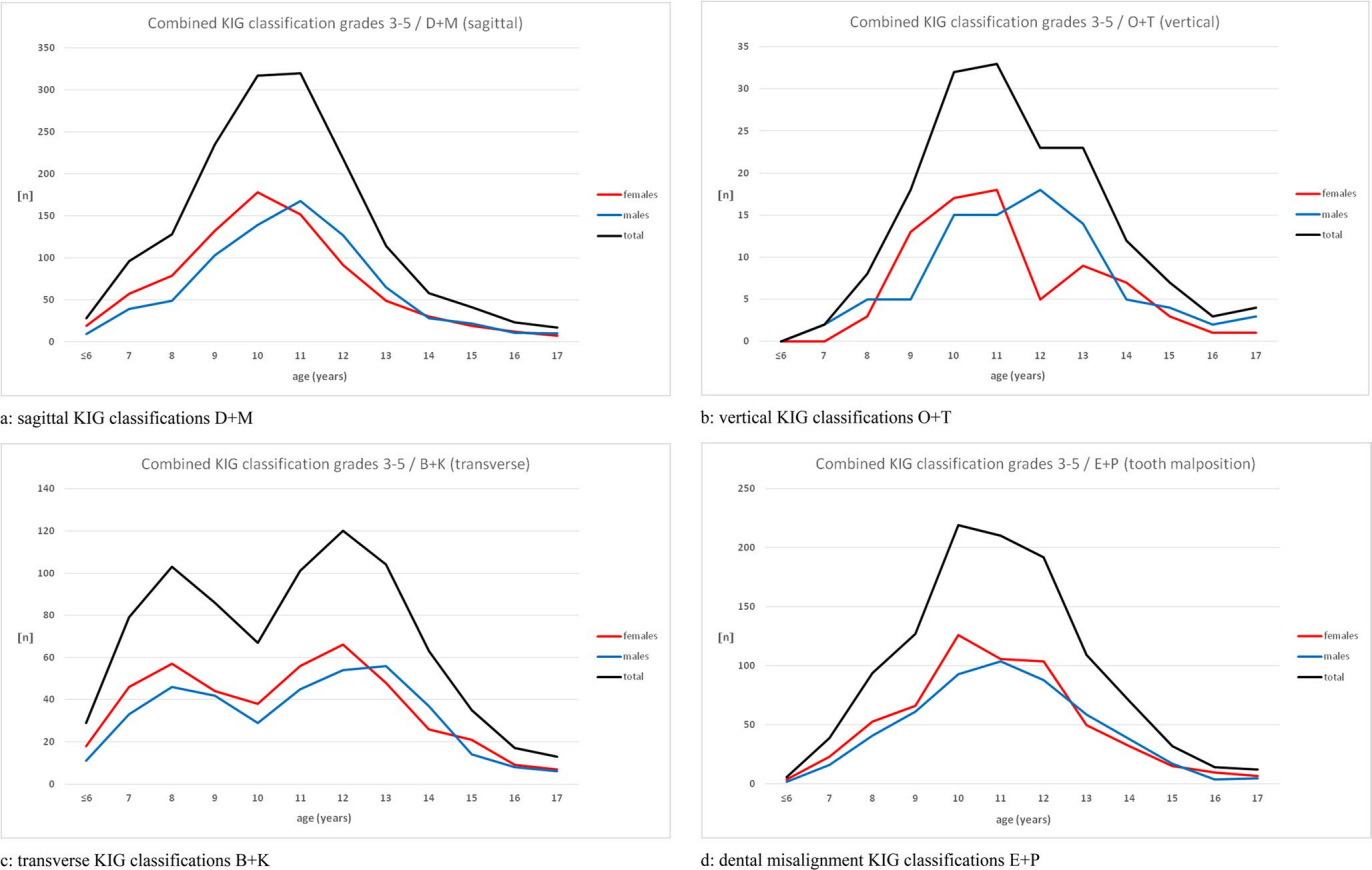
Frequency and age distribution between 2002 and 2021, broken down by combined KIG classifications according to spatial planes and dental malocclusions

The age distribution for the sagittal classifications D + M showed peaks at 10 and 11 years, with highest values in female patients at the age of 10 and in male patients at 11 years (Fig. 4a). The less frequent vertical classifications O + T showed a mean age peak between 10 and 11 years, but when broken down by gender, the highest values were 11 years for girls and 12 years for boys (Fig. 4b). The transverse classifications B + K revealed two age peaks: one for both sexes at around 8 years, and another, more pronounced one at around 12 years of age (peak values for females at 12 and males at 13 years) (Fig. 4c). Patients with the classifications E + P showed an age peak for all patients between 10 and 12 years and, when separated by gender, for female patients at 10 years and male patients at 11 years (Fig. 4d). The age distribution showed significantly different peak values (female patients earlier than male patients) for the classifications D + M, O + T, B + K (at the 2nd age peak) and E + P.

### Frequency and gender distribution of treatment periods regarding spatial plane and abnormal tooth position (Table 5)

#### PG 1 – Early treatment (Table 5)

Out of *n* = 1078 patients in PG 1 in the 20-year-period, *n* = 487 (45.1%) showed sagittal classifications D + M, *n* = 297 (27.5%) transverse classifications B + K, *n* = 266 (24.8%) tooth position deviations E + P, and *n* = 28 (2.6%) vertical classifications O + T.

Female patients predominated the entire cohort and each combined classification: total *n* = 614 f (57.0%)/*n* = 464 m, (43.0%), D + M *n* = 287 f (58.9%)/*n* = 200 m (41.1%), O + T *n* = 16 f (57.1%)/*n* = 12 m (42.9%), B + K *n* = 165 f (55.6%)/*n* = 132 m (44.4%), E + P *n* = 146 f (54.9%)/*n* = 120 m (45.1%).

#### PG 2 – Main treatment (Table 5)

Out of *n* = 1852 patients in PG 2 in the 20-year-period, *n* = 855 (46.2%) had sagittal classifications D + M, *n* = 621 (33.5%) tooth position classification E + P, *n* = 288 (15.5%) transverse classifications B + K and *n* = 88 patients (4.8%) vertical classifications O + T.

Female patients predominated in 2 combinations: E + P *n* = 336 f (54.1%)/*n* = 285 m (45.9%), and B + K *n* = 160 f (55.6%)/*n* = 128 m (44.4%). Male patients predominated in the remaining classifications: D + M *n* = 434 m (50.8%)/*n* = 421 f (49.2%), and O + T *n* = 48 m (54.5%)/*n* = 40 f (45.5%). These results revealed basically no gender differences in this specific cohort (*n* = 957 f, 51.7%/*n* = 895 m, 48.3%).

#### PG 3 – Late treatment (Table 5)

Out of *n* = 771 patients in PG 3 in the 20-year-period, *n* = 253 (32.9%) showed sagittal classifications D + M, *n* = 237 (30.7%) tooth position classifications E + P, *n* = 232 (30.0%) transverse classifications B + K, and *n* = 49 patients (6.4%) showed vertical classifications O + T. Male patients predominated the entire cohort (*n* = 408 m, 52.9%/*n* = 363 f, 47.1%) and each combined classification: D + M *n* = 136 (53.8%) m/*n* = 117 f (46.2%), O + T *n* = 28 m (57.1%)/*n* = 21 f (42.9%), B + K *n* = 121 m (52.2%)/*n* = 111f (47.8%), E + P *n* = 123 m (51.9%)/*n* = 114 f (48.1%).

### Percentage comparison of gender distribution regarding spatial plane and abnormal tooth position in patient groups 1, 2 and 3 (Fig. 5a and b; Table 5)

**Fig. 5:**
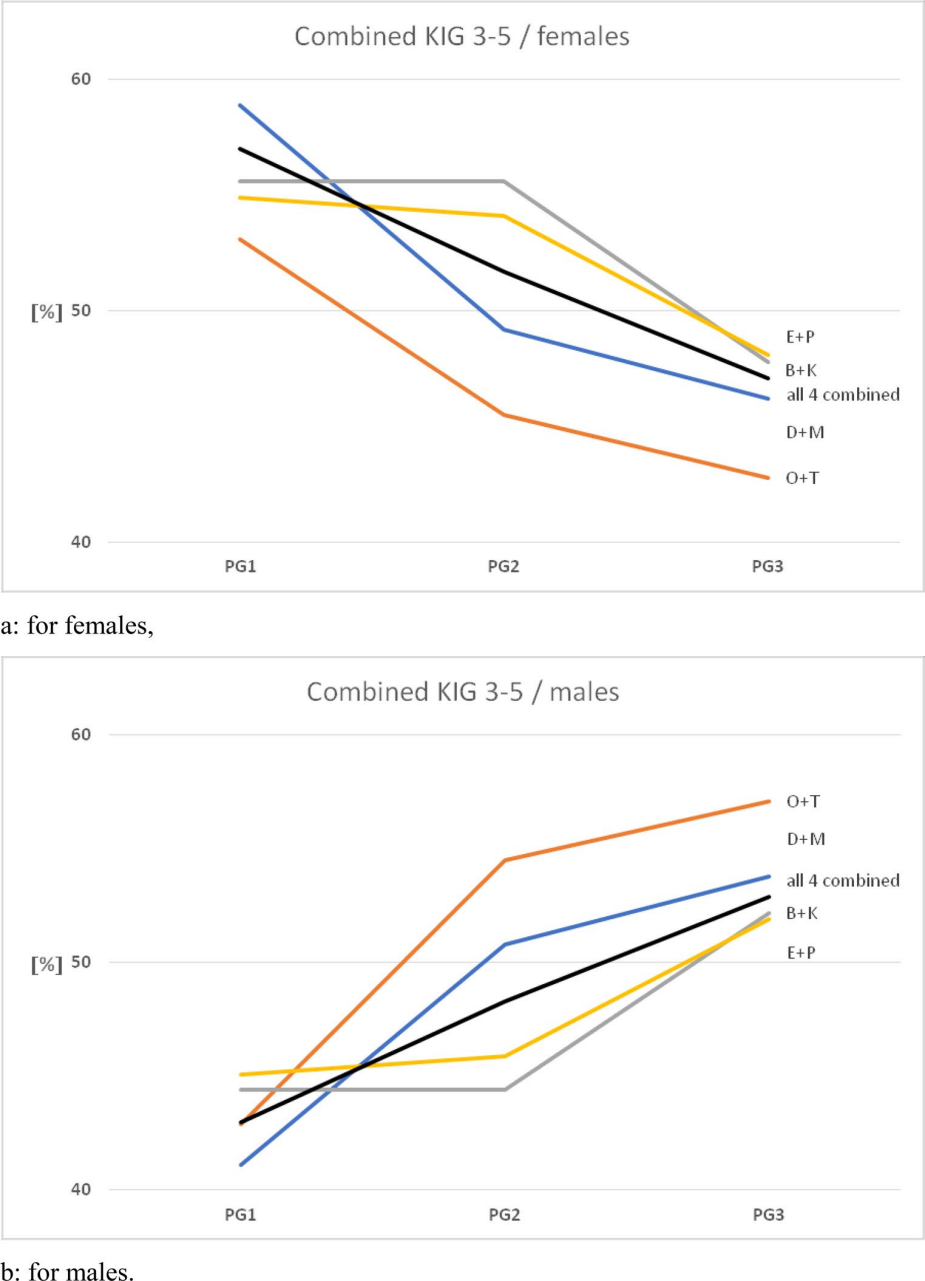
Frequency and gender distribution in the study groups between 2002 and 2021, broken down by combined KIG classifications with grades 3–5 according to spatial planes and dental malocclusions

Figure 5a and b show the contrary development of the percentage share within the different age groups regarding classifications of spatial plane and abnormal tooth position:

In female patients, there is a decrease in PG 3 compared to PG 1 in all patients and all classifications with an almost linear progression in the entire study group. The difference between PG 1 and PG 2 is particularly large for the combined classifications D + M and O + T, and between PG 2 and PG 3 for the combined classifications E + P and B + K.

In male patients, one could observe the reverse: there was an increase in the percentage share in PG 3 compared to PG 1 in all patients and in all combined classifications, with the progress being also almost linear in the total group. The difference between PG 1 and PG 2 in the combined classifications D + M and O + T, and between PG 2 and PG 3 in the combined classifications E + P and B + K was particularly large.

## Discussion

### Collection of data

In this retrospective study, the KIG classification was applied strictly in accordance with the SHI guidelines, so that only the highest possible classification was recorded. The aim was not to draw comprehensive conclusions about the general orthodontic care situation in Germany, since data collection was limited to a single centre. Existing prospective studies by Glasl et al. [2] 2006 and the DMS•6 [4–6] were designed differently. Here, various anomalies of each patient were recorded within narrow age and time limits, although only the DMS•6 was multicentric. Unlike these single-time cross-sectional studies, which were conducted on pre-selected patient groups, the present 20-year cross-sectional study enabled a more differentiated analysis of the gender- and age-dependent prevalence of specific orthodontic anomalies and their combinations for the first time in an unselected clientele.

### Results of the present study

The present 20-year cross-sectional study confirms the results of existing studies [8–10] about the prevalence of individual anomalies and their combinations as well as their age-specific characteristics. In addition, however, gender-specific peculiarities are evident: Predominantly in classifications influenced by growth, there are clear age shifts between the genders at the time of initial diagnosis and thus at the start of treatment. Contrary to that, dental classifications E and P appeared less age- and/or gender-dependent.

It should be noted that the classification-based selective indication for early treatment prior to the late mixed dentition could have led to an increased occurrence of certain anomalies in PG 1. This explains the comparatively higher frequency of the classifications K, D and M in PG 1 and is probably the cause for peaks in K and M at an early age.

This is the first study about the KIG classification system that could prove that gender distribution is fundamentally age-dependent and not anomaly-dependent. The age dependency probably results from females’ earlier growth and maturation. Moreover, certain orthodontic anomalies may become more pronounced with growth and age. To improve differentiation and build on the knowledge gained from earlier studies by Stahl et al. [12] and Grabowski et al. [13], we divided patients into three cohorts based on dentition staging. Still, this remains the first study using the KIG classification system to reveal that gender-specific differences in the incidence of malocclusion are age-dependent and not anomaly-dependent.

This was illustrated by the clear differences between PG 1 (early treatment) with predominance of female patients in 6 individual classifications and all 4 combinations and PG 3 (late treatment) with predominance of male patients in 6 individual anomalies and all 4 combinations. In any case, the effects in the combinations were predominantly caused by the individual anomalies O and K in early treatment and D, B and P in late treatment. Overall, more female patients were diagnosed and treated in 5 out of 8 classifications and in 3 out of 4 classification groups.

### Comparison with existing clinical studies regarding the KIG classification system in Germany

In Germany, only two single-session clinical studies with different study designs exist that have evaluated the prevalence of malocclusions requiring treatment in accordance with the currently valid statutory health insurance guidelines [14]:

In 2004, Assimakopoulou [3] used dental casts in a retrospective setting to investigate the need for orthodontic treatment in *n* = 526 9- to 10-year-old primary school children from Münster, Germany. *N* = 266 of the pupils were male (51%), *n* = 260 (49%) female. The dental casts were analysed using software-based and additional manual measurements. 46% of the study cohort needed treatment according to the KIG classification system.

Glasl et al. [2] analysed the prevalence and development of clinical findings triggering treatment in accordance with the KIG classification in 2004. They studied *n* = 1251 pupils (50.5% male, 49.5% female) aged between 9 and 11 years as part of a school dental examination in Frankfurt am Main, Germany. They identified treatment indication as defined by the statutory health insurance in 41.4% of all study subjects. During this strictly regional study, care was taken to ensure that the children had participated in a preliminary study by Schopf [11] from the year 2000, i.e. prior to the introduction of the KIG classification, wherever possible.

As part of the Sixth German Oral Health Study (DMS•6) [4, 5], a survey of the prevalence of malocclusion in the age group of 8 to 9-year-olds was conducted in 2021 in 16 nationwide study centres [6] with *n* = 705 study participants (51.4% male, 48.6% female). The aim was to represent a nationwide average. The proportion of 8-year-olds was 49.4% and 50.6% of 9-year-olds. The proportion for whom orthodontic treatment is indicated according to the KIG classification was 40.4% in the DMS•6.

When comparing the results of the present study with these single-centre studies conducted in Germany mentioned above [2–5], it must be regarded that the study clientele was always different. The investigated age range was small and the gender distribution potentially pre-selected because of the restriction to school classes in Assimakopoulou [3] and Glasl et al. [2], and to study centres in the DMS•6, where the composition was based on random sampling via residents’ registration offices [4–6].

The present study, like those of Assimakopoulou [3] and Glasl et al. [2], was also regionally limited to a specific catchment area. However, current studies conducted with data from the same practice show that there are no regional peculiarities in KIG findings. Both prevalence and age distribution of KIG classifications with treatment need correspond to the national average [8–10] and may therefore interpreted as representative.

An age-related restriction as in the abovementioned studies [2–5] is not uncritical because certain orthodontic anomalies can become more pronounced with continuing growth and increasing age [12, 13]. This may result in a risk of underestimating the actual prevalence and need for orthodontic care due to the limitations of age-restrictions. Studies with a restricted age range of study subjects are also limited in their informative value regarding the actual prevalence of gender distribution. To avoid this, all patients aged < 18 years and eligible for treatment were included in the present study and divided into 3 age groups that represented decisive phases during the development of the dentition and craniofacial growth. This approach showed not only age-dependent but also gender-specific developments. The latter differed from studies with an age-restricted examination clientele [2–5], in which the percentage of male patients is slightly higher than that of female patients. This may be explained by the preselection occurring when using school classes and study centres. The gender-specific prevalence thus corresponds to the data from the German Federal Statistical Office, according to which more boys than girls have been born every year since at least 1946 [15].

All studies with a narrow age range must therefore be considered limited in their informative value about the actual prevalence of gender distribution. In contrast to the single-centre cross-sectional studies, which were conducted on pre-selected patient groups, the present 20-year cross-sectional study allows a more differentiated view of gender- and age-dependent prevalence of specific orthodontic anomalies and their combinations due to the chosen methodology. It is therefore also a valuable add-on to the results of the DMS•6.

Like this study, the BARMER dental report from June 2024 [16] also revealed an increased number of orthodontic treatments for girls compared to boys. More than *n* = 53,000 patients born in 2005 with statutory health insurance and their orthodontic treatment between 8 and 17 years of age (2013–2022) were analysed. 70% of this group received an initial orthodontic consultation and a cumulative 54.7% received orthodontic treatment. Divided by gender, 60% of all girls and 50% of all boys received orthodontic treatment in the years 2013 to 2022. This gender difference was also evident in all federal states of Germany. The report cites the pursuit of the current ideal of beauty, peer pressure and parental care as possible reasons why girls were treated more frequently than boys [16]. The disadvantage of this finding is that the data was collected cumulatively and, unlike the present study, does not allow any statement to be made about a specific age-dependent prevalence of individual anomalies and their combination in both genders.

### Possible limitations of the study

Although they derive from the ICON, the KIG classifications were not developed to serve as an epidemiological index. Instead, they are a tool used to determine whether patients are eligible for treatment covered by statutory health insurance at various stages of dentition development.

The KIG classifications were performed and recorded by different specialists for orthodontics. According to Gesch et al. [17], there are considerable examiner differences in the classification of subjects into the correct orthodontic indication groups. Different elicitation methods (clinic/model) in the reporting of malocclusion symptoms by several examiners as well as examiners inexperienced in orthodontics may have an unfavourable influence on examiner agreement. For this reason, all KIG classifications and treatment plans were reviewed and validated by another orthodontist applying the four-eye-principle throughout.

## Data Availability

No datasets were generated or analysed during the current study.
